# Genetic Links between Recombination and Speciation

**DOI:** 10.1371/journal.pgen.1006066

**Published:** 2016-06-09

**Authors:** Bret A. Payseur

**Affiliations:** Laboratory of Genetics, University of Wisconsin—Madison, Madison, Wisconsin, United States of America; NIH, UNITED STATES

Meiosis distinguishes cells in the germ line from their somatic counterparts. Part of meiosis involves a bizarre series of events in which cells deliberately damage their DNA and then repair it. Of the many double-strand breaks that are generated, a minority are resolved as reciprocal exchanges between homologous chromosomes [1]. In a wide variety of species, recombination diversifies genomes and is required for the formation of viable gametes. Although the functional role of crossing over should impose strong selective constraints, the rate of recombination varies among individuals. Recombination rate shows resemblance among relatives [2], differs among inbred strains raised in a common environment [3], and responds to artificial selection [4], demonstrating a genetic component to individual rate differences that remains mostly unexplained. A handful of genes responsible for variation in recombination rate have been identified, including modifiers of the total number of crossovers [5–8] and loci that determine the genomic placement of recombination events [9].

As a fundamental part of gametogenesis, meiosis also plays a role in speciation, the means by which one species becomes two. A common observation is that otherwise viable hybrids between genetically differentiated lineages suffer from reduced fertility up to the point of complete sterility—an important reproductive barrier that keeps species distinct [10]. One stage during which problems appear is meiosis, when hybrid gametogenesis sometimes arrests. According to an influential model, hybrid sterility arises from disrupted interactions between different genes that evolved in separate populations [11,12]. The search for incompatibility genes that cause hybrid sterility has so far yielded a short list [13–15].

The fact that only a small number of genes are known to control variation in recombination rate or to contribute to hybrid sterility makes the discovery that one gene mediates both traits especially noteworthy. *Prdm9* encodes a histone methyltransferase that modifies chromatin and is essential for meiosis in mice [16]. Decades of persistent effort from the group of Jiri Forejt demonstrated that incompatibility between *Prdm9* and other loci causes sterility in F_1_ hybrid males formed by crossing two subspecies of house mice [17]. Subsequently, genetic mapping in crosses, population genetic analysis, bioinformatic prediction of DNA binding sites, and association studies jointly identified *Prdm9* as a primary determinant of recombination hotspot location (short stretches of sequence within which most crossovers occur) in mice and humans [9,18–20]. The dual roles of *Prdm9* reveal a tantalizing link between recombination and speciation at the genetic level and motivate the search for other genes that affect both processes.

Now, progress from Jiri Forejt’s lab provides fresh evidence for a genetic connection between recombination and hybrid sterility. Balcova et al. (2016) [21] profile the genome-wide recombination rate by visualizing the immunolocalization pattern of the MLH1 mismatch repair protein that resolves double-strand breaks into crossovers (Fig 1). This powerful approach enables the total number of recombination events to be counted in individual spermatocytes. The authors first use the MLH1 technique to confirm that the genome of an inbred strain representing the house mouse subspecies *Mus musculus musculus* experiences an average of 4.7 more crossovers than the genome of an inbred strain descended primarily from *M*. *m*. *domesticus* (a 19% increase). Next, Balcova et al. (2016) measure recombination rates in a panel of inbred strains, each of which harbors single chromosomes from the *M*. *m*. *musculus* strain on the genomic background of the *M*. *m*. *domesticus* strain. By comparing rates in chromosome substitution strains with the appropriate parental strain, the authors identify three chromosomes that affect the genome-wide crossover number. The locus with the largest phenotypic effect is located on the X chromosome. Rate differences among substitution strains carrying pieces of the chromosome further localize the position of this modifier of the global recombination rate to a 4.7 Mb interval (nicknamed *Meir1* by the authors). This locus lies within a broader part of the X chromosome previously shown to modulate recombination in crosses involving other subspecies of mice [22,23].

**Fig 1:**
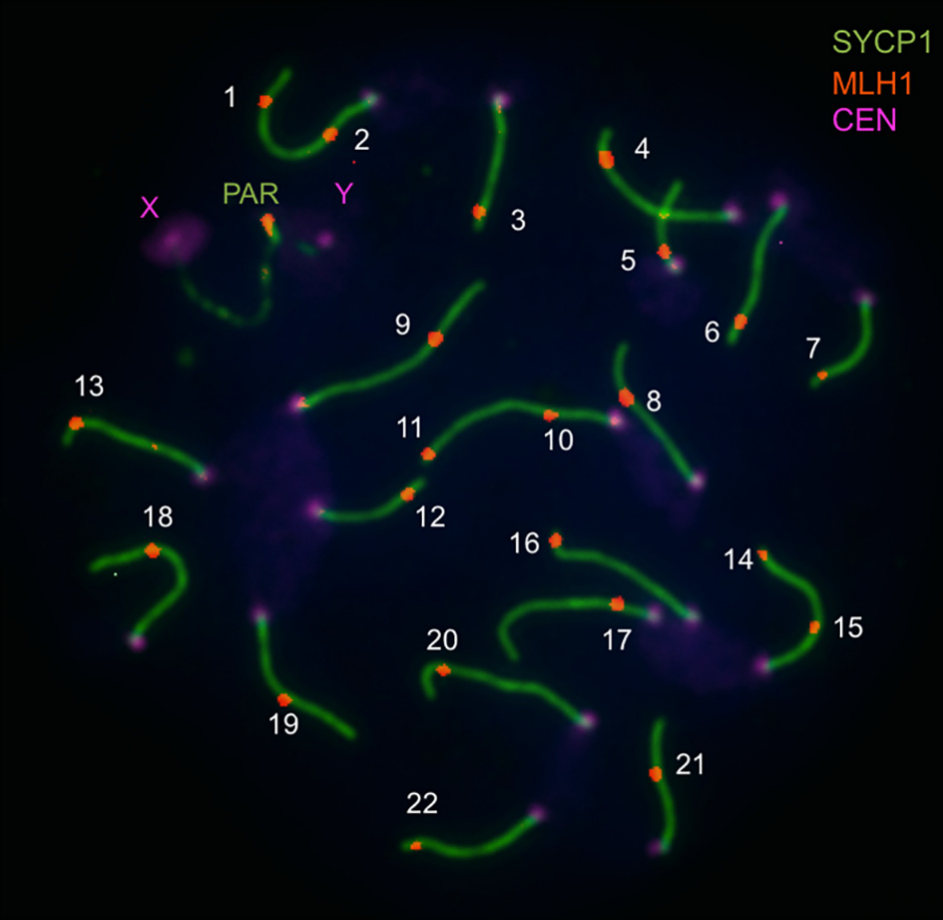
Image illustrating immunofluorescent cytology approach to measuring the genome-wide recombination rate (from Balcova et al., 2016). Pachytene spread of a spermatocyte from the C57/BL6 strain shows central elements of synaptonemal complexes of 19 autosomes and the pseudoautosomal region (PAR) immunostained for SYCP1 (green), MLH1 foci (red), and foci of centromeric proteins (violet). The number of MLH1 foci is used as an equivalent of the number of crossovers.

Several lines of evidence suggest that this region of the X chromosome genetically links recombination and speciation. First, the same interval is associated with multiple correlates of hybrid sterility. Small testes, low sperm count, and morphological abnormalities in sperm observed in F_1_ males from crosses between *M*. *m*. *musculus* mothers and *M*. *m*. *domesticus* fathers all map to this genomic location [24,25]. Second, hybrid male sterility due to one of the loci in this X-linked region (*Hstx2*) involves an incompatibility with *Prdm9* [25]. Finally, the effects of this piece of the X chromosome on recombination and sterility show similar properties, including male specificity.

The findings of Balcova et al. (2016), along with previous discoveries about *Prdm9*, raise the intriguing possibility that recombination and speciation are mechanistically coupled. Remarkably, both the fine-scale placement of crossovers (mediated by *Prdm9*) and the global recombination rate (controlled by *Meir1*) seem to be associated with hybrid sterility. The authors propose that the incompatibility between *Prdm9* and *Hstx2* could inhibit the repair of double-strand breaks, which could in turn lead to the disrupted synapsis of chromosomes and meiotic arrest observed in sterile F_1_ males [26,27]. Regardless of the mechanism, the larger implication is that evolutionary divergence in recombination genes can generate a frequently observed form of reproductive isolation.

Once the causative gene(s) and mutation(s) corresponding to *Meir1* and *Hstx2* are identified in this system, important remaining questions can be answered. Do the joint effects on recombination and hybrid sterility reflect the pleiotropic activities of a single gene or multiple linked genes? What are the molecular, cellular, and developmental bases of these phenotypes? What is the evolutionary history of the causative mutations? Did natural selection drive phenotypic divergence, as seems to be the case for the rapidly evolving *Prdm9* [28]?

The results from Balcova et al. (2016) should also motivate similar studies in other organisms. The observations that recombination rate evolves and that hybrid sterility is a key reproductive barrier are phylogenetically widespread, leaving no obvious reason that the emerging connection between recombination and speciation should be restricted to mice.

## References

[1] Baudat F, de Massy B. Regulating double-stranded DNA break repair towards crossover or non-crossover during mammalian meiosis. Chromosome Res. 2007; 15: 565–577. 17674146 10.1007/s10577-007-1140-3

[2] Kong A, Barnard J, Gudbjartsson DF, Thorleifsson G, Jonsdottir G, Sigurdardottir S, et al Recombination rate and reproductive success in humans. Nat Genet. 2004; 36: 1203–1206. 15467721 10.1038/ng1445

[3] Koehler KE, Cherry JP, Lynn A, Hunt PA, Hassold TJ. Genetic control of mammalian meiotic recombination. I. Variation in exchange frequencies among males from inbred mouse strains. Genetics. 2002; 162: 297–306. 12242241 10.1093/genetics/162.1.297 PMC1462263

[4] Chinnici JP. Modification of recombination frequency in Drosophila. I. Selection for increased and decreased crossing over. Genetics. 1971; 69: 71–83. 5002414 10.1093/genetics/69.1.71 PMC1212690

[5] Kong A, Thorleifsson G, Stefansson H, Masson G, Helgason A, Gudbjartsson DF, et al Sequence variants in the RNF212 gene associate with genome-wide recombination rate. Science. 2008; 319: 1398–1401. 10.1126/science.1152422 18239089

[6] Sandor C, Li W, Coppieters W, Druet T, Charlier C, Georges M. Genetic variants in REC8, RNF212, and PRDM9 influence male recombination in cattle. PLoS Genet. 2012; 8: e1002854 10.1371/journal.pgen.1002854 22844258 PMC3406008

[7] Ma L, O’Connell JR, Van Raden PM, Shen B, Padhi A, Bickhart DM, et al Cattle sex-specific recombination and genetic control from a large pedigree analysis. PLoS Genet. 2015; 11: e1005387 10.1371/journal.pgen.1005387 26540184 PMC4634960

[8] Johnston S, Berenos C, Slate J, Pemberton JM. Conserved genetic architecture underlying individual recombination rate variation in a wild population of Soay sheep (*Ovis aries*). Genetics. 2016; pii: genetics.115.185553. [Epub ahead of print]. 10.1534/genetics.115.185553 PMC4858801 27029733

[9] Parvanov ED, Petkov PM, Paigen K. Prdm9 controls activation of mammalian recombination hotspots. Science. 2010; 327: 835 10.1126/science.1181495 20044538 PMC2821451

[10] Coyne JA, Orr HA. Speciation. Sinauer Associates, Sunderland, MA; 2004.

[11] Dobzhansky T. Genetics and the Origin of Species. Columbia University Press, New York; 1937.

[12] Muller HJ. Isolating mechanisms, evolution and temperature. Biol Symp. 1942; 6: 71–125.

[13] Presgraves DC. The molecular evolutionary basis of species formation. Nat Rev Genet. 2010; 11: 175–180. 10.1038/nrg2718 20051985

[14] Rieseberg LH, Blackman BK. Speciation genes in plants. Ann Bot. 2010; 106: 439–455. 10.1093/aob/mcq126 20576737 PMC2924826

[15] Maheshwari Barbash DA. The genetics of hybrid incompatibilities. Ann Rev Genet. 2011; 45: 331–355. 10.1146/annurev-genet-110410-132514 21910629

[16] Hayashi K, Yoshida K, Matsui Y. A histone H3 methyltransferase controls epigenetic events required for meiotic prophase. Nature. 2005; 438: 374–378. 16292313 10.1038/nature04112

[17] Mihola O, Trachtulec Z, Vlcek C, Schimenti JC, Forejt J. A mouse speciation gene encodes a meiotic histone H3 methyltransferase. Science. 2009; 323: 373–375. 10.1126/science.1163601 19074312

[18] Baudat F, Buard J, Grey C, Fledel-Alon A, Ober C, Przeworski M, et al PRDM9 is a major determinant of meiotic recombination hotspots in humans and mice. Science. 2010; 327: 836–840. 10.1126/science.1183439 20044539 PMC4295902

[19] Myers S, Bowden R, Tumian A, Bontrop RE, Freeman C, MacFie TS, et al Drive against hotspot motifs in primates implicates the PRDM9 gene in meiotic recombination. Science. 2010; 327: 876–879. 10.1126/science.1182363 20044541 PMC3828505

[20] Grey C, Barthès P, Chauveau-Le Friec G, Langa F, Baudat F, de Massy B. Mouse PRDM9 DNA-binding specificity determines sites of histone H3 lysine 4 trimethylation for initiation of meiotic recombination. PLoS Biol. 2011; 9: e1001176 10.1371/journal.pbio.1001176 22028627 PMC3196474

[21] Balcova M, Faltusova B, Gergelits V, Bhattacharyya T, Mihola O, Zdenek T, et al Hybrid sterility locus on chromosome X controls meiotic recombination rate in mouse. PLoS Genet. 2016; 12: e1005906 10.1371/journal.pgen.1005906 27104744 PMC4841592

[22] Murdoch B, Owen N, Shirley S, Crumb S, Broman KW, Hassold T. Multiple loci contribute to genome-wide recombination levels in male mice. Mamm Genome. 2010; 21: 550–555. 10.1007/s00335-010-9303-5 21113599 PMC3002158

[23] Dumont BL, Payseur BA. Genetic analysis of genome-scale recombination rate evolution in house mice. PLoS Genet. 2011; 7:e1002116 10.1371/journal.pgen.1002116 21695226 PMC3111479

[24] Storchova R, Gregorova S, Buckiova D, Kyselova V, Divina P, Forejt J. Genetic analysis of X-linked hybrid sterility in the house mouse. Mamm Genome. 2004; 15: 515–524. 15366371 10.1007/s00335-004-2386-0

[25] Forejt J, Pialek J, Trachtulec Z. Hybrid male sterility genes in the mouse subspecific crosses In: Macholan M, Baird SJE, Muclinger P, Pialek J, editors. Evolution of the House Mouse. Cambridge: Cambridge University Press; 2012.

[26] Bhattacharyya T, Reifova R, Gregorova S, Simecek P, Gergelits V, Mistrik M, et al X chromosome control of meiotic chromosome synapsis in mouse inter-subspecific hybrids. PLoS Genet. 2014; 10: e1004088 10.1371/journal.pgen.1004088 24516397 PMC3916230

[27] Bhattacharyya T, Gregorova S, Mihola O, Anger M, Sebestova J, Denny P, et al Mechanistic basis of infertility of mouse intersubspecific hybrids. Proc Natl Acad Sci U S A. 2013; 110: E468–477. 10.1073/pnas.1219126110 23329330 PMC3568299

[28] Oliver PL, Goodstadt L, Bayes JJ, Birtle Z, Roach KC, Phadnis N. Accelerated evolution of the Prdm9 speciation gene across diverse metazoan taxa. PLoS Genet. 2009; 5: e1000753 10.1371/journal.pgen.1000753 19997497 PMC2779102

